# Multi-omics approach to dissect the significance of lipid metabolism in helper T cell subset

**DOI:** 10.3389/fimmu.2025.1708616

**Published:** 2025-12-10

**Authors:** Toshio Kanno, Keiko Nakano, Yukie Iwao, Yusuke Endo

**Affiliations:** Department of Frontier Research and Development, Laboratory of Medical Omics Research, Kazusa DNA Research Institute, Chiba, Japan

**Keywords:** multi-omics, lipid metabolism, T cell, Acc1, acetyl-CoA carboxylase 1, Th17 and Treg cells, memory T cell

## Abstract

The immunometabolism has fundamentally reshaped our understanding of T cell biology. Recent advances have demonstrated that metabolic reprogramming is not merely a consequence of T cell activation but a central driver of lineage specification and effector function. For example, quiescent naïve T cells primarily rely on mitochondrial oxidative phosphorylation (OXPHOS) and fatty acid oxidation (FAO) to meet baseline energy needs, whereas activation triggers a metabolic shift toward anabolic pathways dominated by aerobic glycolysis and *de novo* biosynthesis of macromolecules. Concurrently, the lipid metabolism confers extensive remodeling: activated T cells upregulate the pathways for *de novo* fatty acid synthesis and cholesterol biosynthesis, uptake, and storage to sustain membrane biogenesis and signal transduction. Conversely, fatty acid catabolism *via* β-oxidation is essential for the generation of memory T cells and the differentiation of regulatory T cells. This review reports recent advances by integrating experimental findings and methodological developments, highlighting how metabolic programs across distinct stages of T cell differentiation—with particular emphasis on the lipid metabolism—govern their specialized functions.

## Introduction

The differentiation of CD4^+^ T lymphocytes into specialized effector subsets, such as Th1, Th2, Th17, regulatory T cells (Tregs), and invariant natural killer T (iNKT) cells, constitutes a central mechanism in adaptive immunity. These distinct lineages play nonredundant roles in pathogen clearance, immune homeostasis, and inflammation. Traditionally, their development has been viewed primarily from the perspective of transcriptional regulation, with lineage-specific master transcription factors, such as T-bet, GATA3, RORγt, and Foxp3, orchestrating subset identity (1–3). However, accumulating evidence has revealed that transcriptional programs alone are insufficient to explain the dynamic and context-dependent nature of T cell differentiation. Instead, a growing paradigm postulates that cellular metabolism acts as an instructive force that integrates extracellular cues to guide T cell fate. This metabolic perspective reshapes the field of immunology by linking nutrient sensing, energy production, and biosynthetic demands with immunological functions.

Several metabolic pathways have been identified as critical determinants of CD4^+^ T cell fate, particularly those involved in fatty acid biosynthesis, oxidative phosphorylation, and glycolysis (4). Most previous studies, however, have focused on phenotypes resulting from genetic deletion of metabolic enzymes, providing limited insight into how the levels and composition of metabolic intermediates are actually altered (5, 6). This gap is particularly evident for lipid metabolites, whose structural complexity have hindered comprehensive analysis at the metabolite level. Lipid metabolism has emerged as a key regulator of T cell responses, with specific lipid metabolites acting as critical metabolic mediators that integrate environmental cues with intracellular signaling processes (7). Furthermore, RORγt, a well-known transcription factor to play crucial roles in thymic development, lymph node organogenesis, and Th17 differentiation, recognizes lipid-ligands for full activation (4). Against this background, lipid metabolism has attracted increasing attention within the field of immunometabolism. Importantly, recent advances in immunometabolism and mass spectrometry technologies have begun to enable detailed profiling and structural characterization of lipid metabolites that modulate immune function. Furthermore, the integration of metabolomic data with gene and protein expression analyses enables the identification of which metabolic enzymes underlie the observed changes in metabolite levels. Mapping the metabolic pathway would provide deeper insights into how specific metabolic processes contribute to the regulation of immune cell function.

Central to lipid metabolic network is the mechanistic target of rapamycin (mTOR), which integrates environmental cues, such as cytokine signaling, nutrient availability, and antigen stimulation to coordinate cellular growth and energy use (8–10). The downstream effectors of mTOR, including sterol regulatory element-binding proteins (SREBPs) and peroxisome proliferator-activated receptor gamma (PPARγ), activate transcriptional programs involved in the lipid uptake and biosynthesis (11, 12). Among biosynthetic enzymes, acetyl-CoA carboxylase 1 (ACC1), a rate-limiting enzyme in *de novo* fatty acid synthesis, has emerged as a pivotal modulator of CD4^+^ T cell differentiation and memory cell formation (13, 14). These insights have been largely enabled by the application of multi-omics approaches, particularly transcriptomics, proteomics, and metabolomics, which permit comprehensive profiling of the gene expression, protein abundance, and metabolite flux at both bulk and single-cell resolution (1, 15–17). Integrative analyses using these technologies have also revealed striking discrepancies between the mRNA and protein levels. These observations highlight the importance of post-transcriptional and post-translational regulation of T cell activation and lineage commitment (18, 19).

In this review, we summarize the recent findings that elucidate how the lipid metabolism and its regulatory enzymes govern the differentiation, survival, and memory potential of CD4^+^ T cells. Furthermore, we highlight how integrative multi-omics approaches have uncovered previously unappreciated roles of metabolic circuits in shaping the T cell function. We further describe how metabolic reprogramming, induced by signals such as TCR activation and the cytokine milieu, intersects with lineage-specific transcriptional programs to define effector and regulatory phenotypes. Specific attention is given to the mechanisms by which *de novo* fatty acid synthesis and sphingolipid metabolism modulate the development of Th17, Treg, and iNKT cells, and memory T cell formation. By evaluating both experimental insights and methodological advances, this review aims to provide a comprehensive perspective on the emerging field of the T cell lipid metabolism and identify key challenges and future directions in the integration of metabolic data into immune cell biology.

## Dissection of the T cell function through transcriptomic, proteomic, and post-translational analyses during differentiation and activation

**Figure 1:**
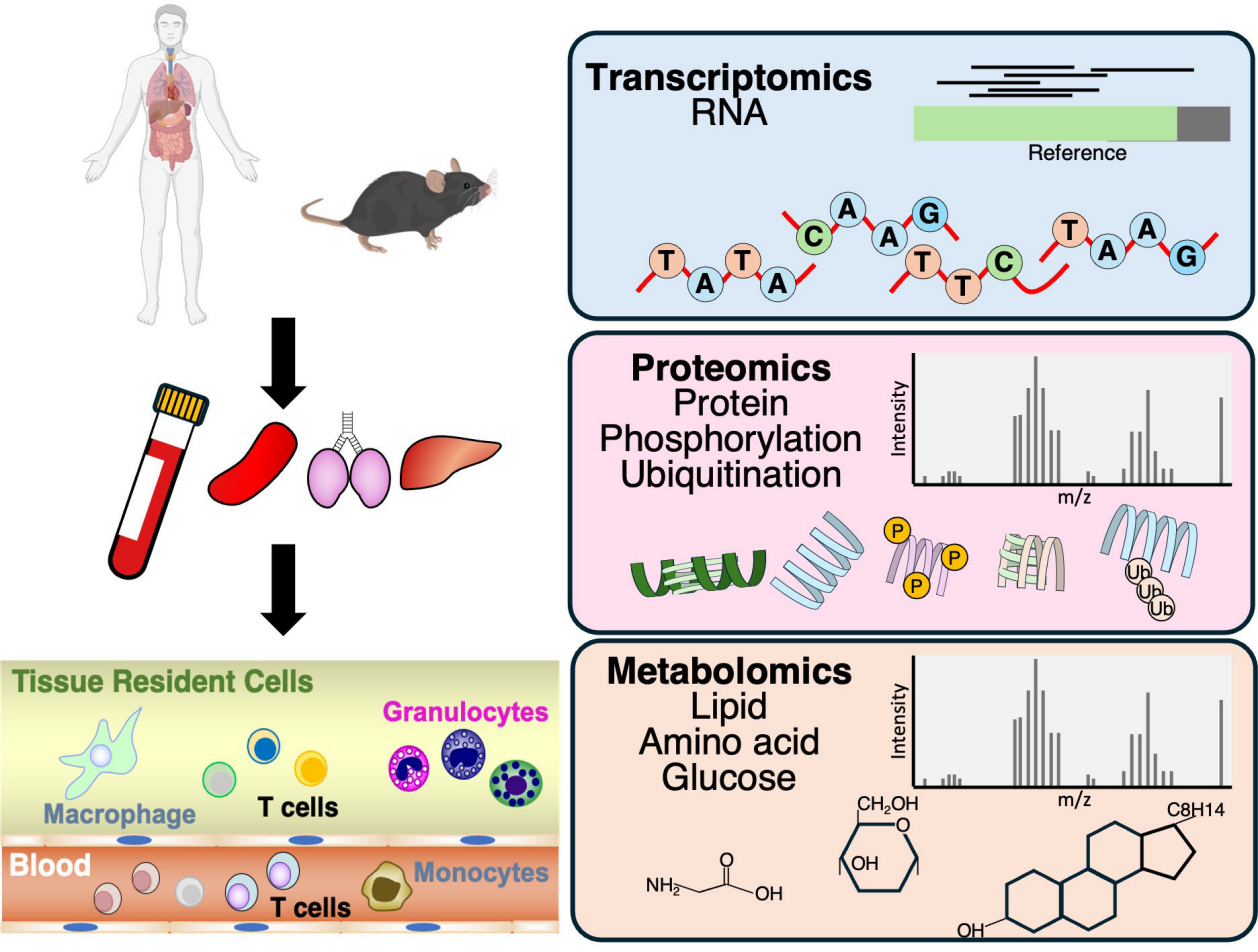
Schematic representation of the multi-omics approach integrating transcriptomics, proteomics, and metabolomics. T cells differentiated *in vitro* and those generated directly *in vivo* are collected and subjected to high-throughput data acquisition, including next-generation sequencing for transcriptome profiling, as well as mass spectrometry-based proteomics and metabolomics. Cross-platform data integration enables identification of key molecular signatures, functional pathways, and regulatory networks, providing a comprehensive view of the biological system under investigation.

Omics analyses have gained increasing attention as a powerful tool for characterizing the transcriptomic, proteomic, and metabolomic landscapes of immune cells (15, 16) (**Figure 1**). Early studies focused on cultured cells or whole-organ samples; however, advances in instrumentation and methodology now allow high-precision and in-depth profiling of primary cells. Transcriptome analyses using next-generation sequencing (NGS) have become highly prevalent, driven by reduced costs and the value of comprehensive gene expression profiling (1, 17). Gene expression profiling has provided key insights into the development and maintenance of effector Th cells and memory T cells, including stem cell–like memory T cells (T_SCM_), central memory T cells (T_CM_), Effector memory T cells (T_EM_), and tissue-resident memory (T_RM_) T cells (1, 17, 20). Effector Th cell subsets are characterized by lineage-specific cytokines and transcription factors. Beyond these classical markers, transcriptome-wide analyses have demonstrated that effector Th subsets can be clearly distinguished based on their global gene expression profiles (1, 17). Memory T cells are broadly classified into distinct subsets based on their differentiation status, migratory capacity, and functional potential. T_SCM_ represent the least differentiated population, characterized by a naïve-like phenotype (CD45RA^+^CCR7^+^CD62L^+^) and self-renewal capacity, yet capable of generating all other memory and effector subsets. T_CM_ express CCR7 and CD62L, allowing recirculation through secondary lymphoid organs, and exhibit high proliferative potential with rapid cytokine production upon reactivation. T_EM_ lack CCR7 and CD62L, reside mainly in peripheral tissues and circulation, and exert immediate effector functions such as cytokine secretion. In contrast, T_RM_ are non-circulating cells that permanently reside within peripheral tissues (e.g., lung, skin, gut), identified by expression of CD69 and CD103, and provide rapid on-site immune protection. Together, these subsets form a hierarchical and functionally diverse memory T cell pool that underpins long-term immune surveillance and recall responses.

Hierarchical clustering analyses of the global gene expression revealed that Th17 and iTreg cells are transcriptionally distinct from Th1 and Th2 cells in both *ex vivo-* and *in vitro*-generated effector Th subsets (1, 17). Since TGF-β is required for the differentiation of Th17 and iTreg cells, TGF-β may contribute to their distinct expression signatures. In addition to the traditional bulk RNA-seq analysis of cell populations, single-cell RNA sequencing (scRNA-seq) has emerged as a systematic and efficient approach to profile transcriptomes at single-cell resolution. This approach has identified a memory precursor-enriched population (ACC1^lo^CCR7^hi^CD137^lo^Blimp1^lo^TCF1^hi^) during the early differentiation phase of effector CD4^+^ T cells (13). CD4^+^ T cells with the phenotype from the spleen and lung exhibited a robust and significant increase in memory cell formation relative to the ACC1^hi^CCR7^lo^CD137^hi^Blimp1^hi^TCF1^lo^ population. Furthermore, a combination of bulk RNA-seq and scRNA-seq analyses revealed heterogeneity among established CD4^+^ Trm cells from LCMV-infected mice (20). At one week post-infection, gut-resident CD4^+^ T cells displayed a similar gene expression profile to that of splenic Th1 cells. However, after three weeks, these cells acquired a distinct transcriptional signature that differed from both Th1 and Tfh cells. This study also showed that Bcl6 is required to support the small intestine CD4^+^ Trm program, likely by maintaining the memory qualities of long-lived Trm populations.

In addition to transcriptomic analyses, there has been a growing trend toward comprehensive profiling of proteins, focusing on primary immune cells. For example, a quantitative proteomic analysis revealed that after 24 h of TCR stimulation, thousands of proteins undergo expression changes in both CD4^+^ and CD8^+^ T cells (21). The quantitative assessment of protein copy numbers has allowed for the global characterization of the pathways related to cell cycle progression, translational machinery, and metabolism, including glucose transport, the mitochondrial function, and the fatty acid metabolism. Notably, a marked increase in proteins associated with the energy sensor mTORC1 was observed, and its inhibition led to divergent outcomes in naïve and effector T cells (21). While mTORC1 inhibition impairs cell cycle progression in stimulated naïve T cells, but not in effector cells, it consistently alters the metabolism in both, suggesting a context-dependent role for mTORC1 during T cell responses. Furthermore, this study showed that the ribosome content of T cells increased approximately 10-fold upon activation, accompanied by strong upregulation of eukaryotic initiation factor 4F (eIF4F), a key regulator of mRNA translation. Interestingly, eukaryotic translation initiation factor 4E-binding protein (4-EBP), which is a translation repressor, was detected only after T cell activation and it reached approximately 20,000 copies. In contrast, its target eIF4F reached >500,000 copies after activation. Therefore, this study highlights the importance of the quantitative ratio of 4-EBP1 to eIF4F when evaluating the impact of 4-EBP-mediated translational repression (21).

Beyond evaluating the expression of proteins, proteomic approaches now allow the comprehensive analysis of post-translational modifications, including phosphorylation and ubiquitination, which are dynamically regulated by TCR signaling (18, 19). The protein expression showed no significant changes between 2 and 8 h after TCR stimulation, but it underwent pronounced changes from 16 h (18, 19). In contrast, the phosphorylation of AKT1, ARAF, and GSK3β was elevated at 2 h (18). Subsequently, the phosphorylation of MYC, MAPK, and 4-EBP1 increased between 8 and 16 h (18). Although 3,280 peptides were phosphorylated after TCR stimulation, the timing and duration of these modifications differed. Among these, 721 peptides showed transient phosphorylation within 16 h and subsequently returned to levels comparable to those before activation. A previous report has shown that Raptor, a key component of mTORC1, functions as a central regulator of CD4^+^ and CD8^+^ T cell exit quiescence (22). Raptor deficiency impairs cell activation and proliferation (22). Combined proteomic and phosphoproteomic analyses revealed that Raptor regulates the expression of 1,326 proteins and the phosphorylation of 547 peptides, including well-established mTORC1 substrates such as RPS6, CAD, and 4EBP1 (18, 19).

Another study demonstrated that the TCR re-stimulation of activated CD4^+^ T cells triggers the ubiquitinome program (19). Ubiquitination can mediate both the degradative and nondegradative pathways. Lysine 48 (K48)-mediated polyubiquitination typically targets proteins for proteasomal degradation, whereas monoubiquitination, multi-monoubiquitination, and ubiquitin modifications at lysines other than K48 regulate non-degradative processes. Approximately 600 proteins exhibited an altered ubiquitination status upon TCR re-stimulation, including changes in the modification intensity or ubiquitin chain type. Notably, approximately 25% of these modifications were observed exclusively in pre- or re-stimulated cells (19). This study also revealed the prevalence of non-degradative ubiquitination in re-stimulated T cells, particularly at K29, K33, and K63, which are known to regulate key cellular processes, such as signal transduction, protein trafficking, and complex assembly, rather than targeting proteins for degradation (19). These findings align with multiple reports highlighting the importance of non-degradative ubiquitination for signal transduction in TCR stimulation (23–25). It has been reported that K33-meditaed polyubiquitination of TCRζ modulates tyrosine kinase ZAP-70 activation (24). Furthermore, the proteolysis-independent ubiquitination of the p85 subunit of PI3K affects its recruitment to CD28 and TCRζ (25). Non-degradative ubiquitination also regulates the differentiation of effector Th cell subsets (26–28). The E3 ubiquitin ligase Hectd3 is necessary for pathogenic Th17 cell generation in experimental autoimmune encephalomyelitis (EAE) through STAT3 and MALT1 (26). Hectd3 promotes non-degradative K27 polyubiquitination at STAT3 K180, which is essential for robust generation of RORγt^+^IL-17A^hi^ Th17 cells. K27 and K29 polyubiquitination of MALT1 K648 is critical for NF-κB activation and the Th17 cell function (26, 27). The deletion of TRAF6 in Tregs impairs the suppressive function, immune homeostasis, and antitumor immunity in mice (28). TRAF6‐mediated K63‐ubiquitination promotes the nuclear accumulation of FOXP3, thereby sustaining Treg functions.

The development of proteomics has enabled comprehensive analysis of protein expression, allowing more accurate inference of cellular functions that are difficult to assess based on gene expression alone. In addition, the adoption of integrated omics approaches, including post-translational modifications, has advanced our understanding of biological phenomena that cannot be explained solely by protein expression. By analyzing proteins that undergo post-translational modifications with simultaneous assessment of whole protein expression, it is possible to identify upstream factors that regulate post-translational modifications (18, 19). It also allows systematic mapping of downstream proteins affected by these post-translational modifications. As these integrated analyses advance, it is now possible to move beyond a single-time-point snapshot of protein expression or modification and begin to predict the dynamic behavior of pathways, such as which will become activated or degraded. In the following section, we focus on examples of immune cell studies using integrated omics and highlight insights revealed through these comprehensive analyses.

## Integrative omics reveals hidden layers of T cell metabolic regulation

While transcriptomic analyses have long been the foundation of immunological research, growing evidence indicates that the expression of mRNA alone provides an incomplete picture of the cellular state and function. In T cells, discrepancies between transcript and protein levels are frequently observed during activation and differentiation, partly because of the widespread influence of post-transcriptional, translational, and post-translational regulation (2). For example, recent proteomic analyses of CD4^+^ T cell subsets revealed that TCR activation altered the expression of nearly half of both the transcriptome and proteome, but with a surprisingly low correlation between RNA and protein abundance across genes (2, 29, 30). These results suggest that protein-level measurements are essential to accurately interpret cellular responses, particularly during rapid and context-dependent transitions such as effector differentiation. Furthermore, the use of data-independent acquisition mass spectrometry (DIA-MS) and parallel RNA sequencing from the same cell population has enabled simultaneous quantification of over 10,000 transcripts and 8,000 proteins, thus offering a uniquely comprehensive view of the molecular landscape driving T cell fate decisions (29, 30). Their analysis revealed that nearly 30% of the genes showed either a weak or no correlation between the transcript and protein levels, particularly among immune-related molecules and metabolic enzymes (1). This discordance was especially prominent in transcription factors and surface markers that define T cell subsets (1). For instance, lineage-defining transcription factors, such as *Tbx21* (T-bet), *Rorc* (RORγt),a and *Foxp3* display delayed or dampened protein expression relative to their mRNA kinetics (1). Similar discrepancies between mRNA and the protein expression levels have been observed in human primary T cells (31). This inconsistency suggests that despite a marked increase in mRNA transcription, the corresponding protein levels may lag due to limitations in ribosome abundance or translational activity (31). These findings emphasize the importance of measuring the proteomic output of cells to understand their current functional state and developmental potential. By resolving these RNA-protein mismatches, proteomics provides a critical layer of biological interpretation that complements transcript-based approaches, particularly in rapidly changing cellular contexts, such as T cell activation and differentiation. Furthermore, the integration of RNA and protein analyses provides deeper insight into gene regulation. For example, when changes are observed in both protein and RNA, this may indicate that the gene is under more active regulation. This trend was observed for subset-specific T cell cytokines such as IFN−γ, IL−4, IL−17A, and IL−17F (1). If changes are observed only at the protein level, this may suggest that the proteins are highly stable, have a long half-life, or are regulated via post-translational modifications (32). Conversely, if expression changes are detected only at the RNA level, it may indicate that the corresponding protein expression could increase subsequently (33). Such integrated analyses of protein and RNA are therefore valuable for inferring properties of the expression machinery that cannot be discerned from expression levels alone.

**Figure 2:**
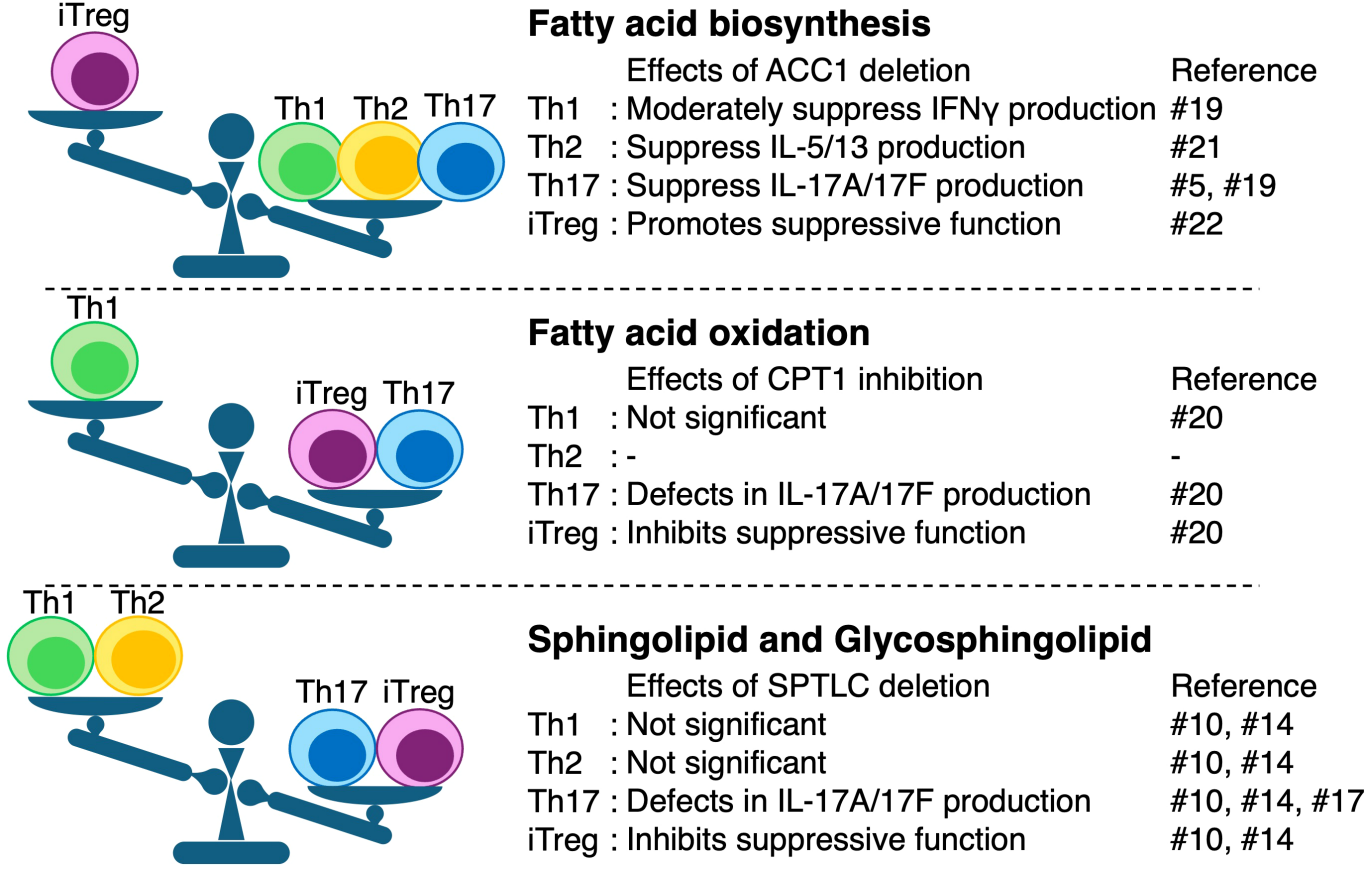
Functional impact of metabolic enzyme perturbation across distinct lipid metabolic pathways in T cell subsets. This figure summarizes effects of genetic deletion or pharmacological inhibition of key enzymes in fatty acid biosynthesis (ACC1), fatty acid β-oxidation (CPT1), and sphingolipid/glycosphingolipid synthesis (SPTLC1). Multi-omics analyses reveal that sphingolipid metabolism provides an additional layer of functional segregation among T cell subsets. No relevant studies on fatty acid oxidation (FAO) in Th2 cells could be identified, and therefore it is not discussed here.

Previous studies have well investigated the importance of ACC1 in T cell biology; however, since ACC1 is the rate-limiting enzyme of fatty acid synthesis, it remains unclear which specific lipid metabolites are most relevant (**Figure 2, upper**). Both metabolomic and lipidomic profiling have further extended our understanding of T cell biology by revealing subset-specific metabolic signatures that are not detectable at the transcript or protein levels alone (**Figure 2**). In particular, recent untargeted metabolomics analyses coupled with proteomics have uncovered the dynamic remodeling of cellular lipids during T cell activation and effector differentiation (2, 34–36). Using high-resolution mass spectrometry, we demonstrated that the biosynthesis of sphingolipids, complex lipids involved in membrane integrity and signal transduction, is significantly upregulated in all major effector T cell subsets, including Th1, Th2, Th17, and iTreg cells (2). This study evaluated the early metabolic changes during Th cell differentiation by stimulating naïve T cells through TCR signaling and culturing them with subset-specific cytokines. Proteomic analysis revealed that following 48 h of TCR stimulation, these Th subsets consistently upregulated the protein expression of key lipid biosynthetic enzymes, including ACC1, FASN, SCD1/2, FADS1/2, ELOVL1/6, and ACSL3–6. Untargeted lipidomics also showed that approximately 500 lipid species, among 567 detected lipid species, increased more than 2-fold in each T cell subset. Interestingly, enzymes involved in ceramide biosynthesis, such as CERS5 and DEGS1, were commonly upregulated across all subsets. In accordance with these observations, there were changes in the amounts of hexosyl ceramides (HexCer) and di-hexosyl ceramides (DiHexCer), which are glycosphingolipids. The species composed of d18:1 and C16:0 increased in both HEX-Cer and DiHEX-Cer, whereas those composed of d18:1 and C24:1 were elevated in Cer, HEX-Cer, and DiHEX-Cer. PCA further revealed that both lipid metabolites and lipid metabolism-related proteins in Th17 and Treg cells differed from those in Th1 and Th2 cells. Notably, Th17 and iTreg cells exhibit an enhanced glycosphingolipid metabolism among effector Th subsets. The pharmacological inhibition of this pathway selectively suppresses their differentiation without affecting Th1 or Th2 development by reducing the expression of lineage-specific transcription factors such as RORγt and Foxp3 (2).

Importantly, even in humans, Th17 and iTreg cells exhibit elevated sphingolipid and glycosphingolipid metabolism compared to Th1 and Th2 cells (34). These findings indicate that distinct T cell lineages utilize specialized lipid biosynthetic programs to support their effector functions and lineage stability. Many studies in both human and murine T cells have highlighted the importance of fatty acid metabolism, indicating a critical role for fatty acid biosynthesis in Th1, Th2, and Th17 cells, and an essential function of fatty acid oxidation in Treg cells (**Figure 2, middle**) (14, 37–41). Nevertheless, owing to the complexity of fatty acid metabolic pathways, it remains unclear which specific fatty acid species modulate the T cell function. In this context, the sphingolipid metabolism has emerged as a key regulator of the T cell subset function. These findings underscore the importance of lipidomics in identifying lineage-specific metabolic vulnerabilities and suggest new opportunities for subset-targeted immunomodulation.

Moreover, the integration of metabolomic and proteomic data revealed that alterations in lipid content were not merely correlative, but functionally instructive (16, 36). This lipid dependence may reflect the unique membrane and signaling demands of certain subsets. For example, glycosphingolipids serve as structural scaffolds for immunological synapse formation and cytokine receptor signaling, which are particularly critical for Th17 and iTreg cell differentiation. In accordance with these observations, Thiruvaimozhi et al. showed that deletion of serine palmitoyltransferase long chain base subunit 1 (SPTLC1), which catalyzes the rate-limiting step in the *de novo* synthesis of sphingolipids, specifically in CD4^+^ T cells, represses the pathogenesis of EAE (**Figure 2, lower**). Loss of SPTLC1 causes aberrant ROS production, thereby inhibiting IL-17A production (42). Liver X receptor β (LXRβ) may partially account for the regulation of sphingolipid and glycosphingolipid biosynthesis (43). T cells predominantly express LXRβ, but not LXRα, and stimulation with an LXR agonist leads to the upregulation of UDP-glucose ceramide glucosyltransferase (UGCG), a rate-limiting enzyme in glycosphingolipid synthesis. LXRβ stimulation also increased the production of glycosphingolipids, such as HexCer. Since metabolites are dynamically and rapidly converted, analyzing the expression of metabolic enzymes at the protein or RNA level alone is insufficient (44). Thus, as demonstrated in these studies, the integration of metabolomic and proteomic analyses enables the identification of which enzyme expression changes are responsible for the observed alterations in metabolite levels. In addition, enzymatic activity is also regulated by post-translational modifications such as phosphorylation and ubiquitination. Given the recent advances in omics analyses of post-translational modifications, further integration with lipidomics is expected to enable the construction of even more detailed and multilayered metabolic maps in the future.

Most omics analysis approaches extract cells from their natural context and, thus, lack spatial information. Spatial scRNA-seq is a powerful technique that captures the transcriptome of individual cells while retaining their spatial context within a tissue. Trm, which are considered a highly active subset within the memory T cell population, have been characterized through the following experimental approaches: labeling methods based on intravenous injection of fluorescent antibody, expression of surface markers including CD69 and CD103, and parabiosis experiment. However, these analyses discard the information of the localization of Trm cells within tissues or the kinds of neighboring cells. Through spatial scRNA-seq of Trm cells, recent research has revealed that the signaling of the intestinal architecture supports two distinct Trm cell states: differentiated Trm cells and progenitor-like Trm cells, located in the upper villus and lower villus, respectively (45). This diversity is mediated by distinct ligand–receptor activities, cytokine gradients and specialized cellular contacts. Especially, *Cxcl9* and *Cxcl10* signals were heavily enriched in the top half of the lamina propria. Deletion of CXCR3, a receptor of these chemokines, decrease the accumulation of T cells in the lamina propria, lower villus area and muscularis. These chemokines signaling might ultimately condition the destination of Trm cells in the tissue. In addition to gene expression, analyses incorporating spatial information have been applied to metabolite profiling (46). The matrix-assisted laser desorption/ionization (MALDI) mass spectrometry imaging have revealed the distinct spatial distribution of lipid metabolites in colorectal cancer. MALDI imaging shows that the phosphatidylcholine (PC [37:5]) was most abundant in the stromal–immune compartment and phosphatidylinositol (PI [34:1]) predominantly in cancer cells. An advantage of this approach is that immune cells can be labeled with antibodies, allowing the lipid metabolite profiles of individual cells to be evaluated. CD204^+^ macrophages also displayed a distinctive lipid profile, with phosphatidylglycerol (PG [40:7]) and lysophosphatidylinosytols (LPI [18:1]) being more abundant compared to T cells, B cells, and monocytes. Interestingly, LPI (18:1) has been suggested to play a role in cancer by inducing ERK1/2 phosphorylation via the GPR55 receptor (47, 48). In the future, integrating such spatial information with analyses of immune cells and lipid metabolites is expected to enable detailed investigations of immune cell function and dynamics *in vivo*. Especially, because immune responses are influenced by cell–to–cell interactions and the immune microenvironment niche, spatially resolved single-cell analyses have the potential to uncover biological phenomena that conventional expression or metabolite analyses may have missed.

Taken together, integrative omics approaches have profoundly expanded our understanding of T cell biology by revealing the molecular layers that are invisible to single-modality analyses. These technologies have uncovered critical disconnects between the expression of mRNA and proteins, identified subset-specific metabolic dependencies, and uncovered previously unappreciated lipid-mediated regulatory circuits, such as sphingolipid pathways essential for Th17 and iTreg differentiation. Furthermore, single-cell resolution and multilayered data integration now enable the mapping of transitional states, metabolic checkpoints, and fate decisions with high precision. In the following sections, we explore how these mechanistic insights converge on the broader themes of immune regulation, clinical translation, and future research directions. Particular attention has been given to the therapeutic modulation of T cell metabolism and the challenges associated with integrating omics data into predictive and actionable frameworks.

## Lipid metabolic control of T cell differentiation

Upon activation, T cells reprogram their metabolic pathways to meet the bioenergetic demands for rapid proliferation. The process towards an anabolic phenotype during T cell activation is regulated by several signaling pathways and transcription factors. T cell stimulation via CD28 co-stimulation with T cell receptor engagement drives rapid proliferation through activation of the PI3K–Akt and mTOR pathways (3, 12). Within the first 24–30 h of anti-TCR/CD28-mediated stimulation, T cells generate markedly increased amounts of metabolites involved in the anabolic pathways of glucose, amino acids, polyamines, carbohydrates, nucleotides, and lipids (49, 50). TCR stimulation–dependent metabolic reprogramming is accomplished through dynamic changes in the expression of metabolic enzymes downstream of mTOR activation (49, 50). In some contexts, mTOR controls glucose metabolism via modulation of hypoxia-inducible factor 1 subunit alpha (Hif-1α) (8, 10, 51, 52). The genetic deletion of Hif-1α in Th17 or activated CD8^+^ T cells, but not in other T cell subsets, leads to a reduction in the expression of Glut1 and other metabolic genes involved in glycolysis (8, 10, 51, 52).

In addition to the glucose metabolism, other metabolic pathways such as lipid metabolism are now recognized as critical aspects of T cell activation. For example, T cells deficient in Raptor, an essential component of mTORC1, exhibit impaired lipid biosynthesis accompanied by diminished protein levels of two primary regulators of lipid biosynthesis: SREBP1 and SREBP2 (11, 22, 53). SREBPs play an essential role in metabolic reprogramming in response to mitogenic stimulation of CD8^+^ T cells (11). Kidani et al. reported that inactivation of SREBPs *via* genetic deletion of the SREBP cleavage-activating protein (SCAP) results in diminished T cell proliferative capacity both *in vitro* and *in vivo* during viral infection (11). They also demonstrated that the functional loss of SREBPs not only attenuates lipid synthesis, but also reduces the prevalence of metabolic reprogramming towards a glycolytic phenotype in activated CD8^+^ T cells. Notably, the SREBP activity is not required for the homeostatic proliferation of naïve or memory CD8^+^ T cells (11). Together, these findings suggest that the lipid biosynthesis pathways modulate mTOR-mediated metabolic reprogramming during T cell activation.

Our group has reported that activated CD4^+^ T cells utilize exogenous FAs as nutrients via the upregulation of PPARγ and its target genes, which are associated with the uptake of FAs (12). Unsaturated fatty acids and other fatty acid metabolites such as 15-deoxy-Δ12,14-prostaglandinJ2 are suspected ligands of PPARγ in some cell types, including adipocytes (54). Therefore, these FA metabolites may bind to PPARγ and control target genes involved in the uptake of FA in activated CD4^+^ T cells. Thus, both the uptake of FA and the biosynthesis pathways are required for the early and full activation of naïve or memory CD4^+^ T cells in response to TCR stimulation in both murine and human systems.

**Figure 3:**
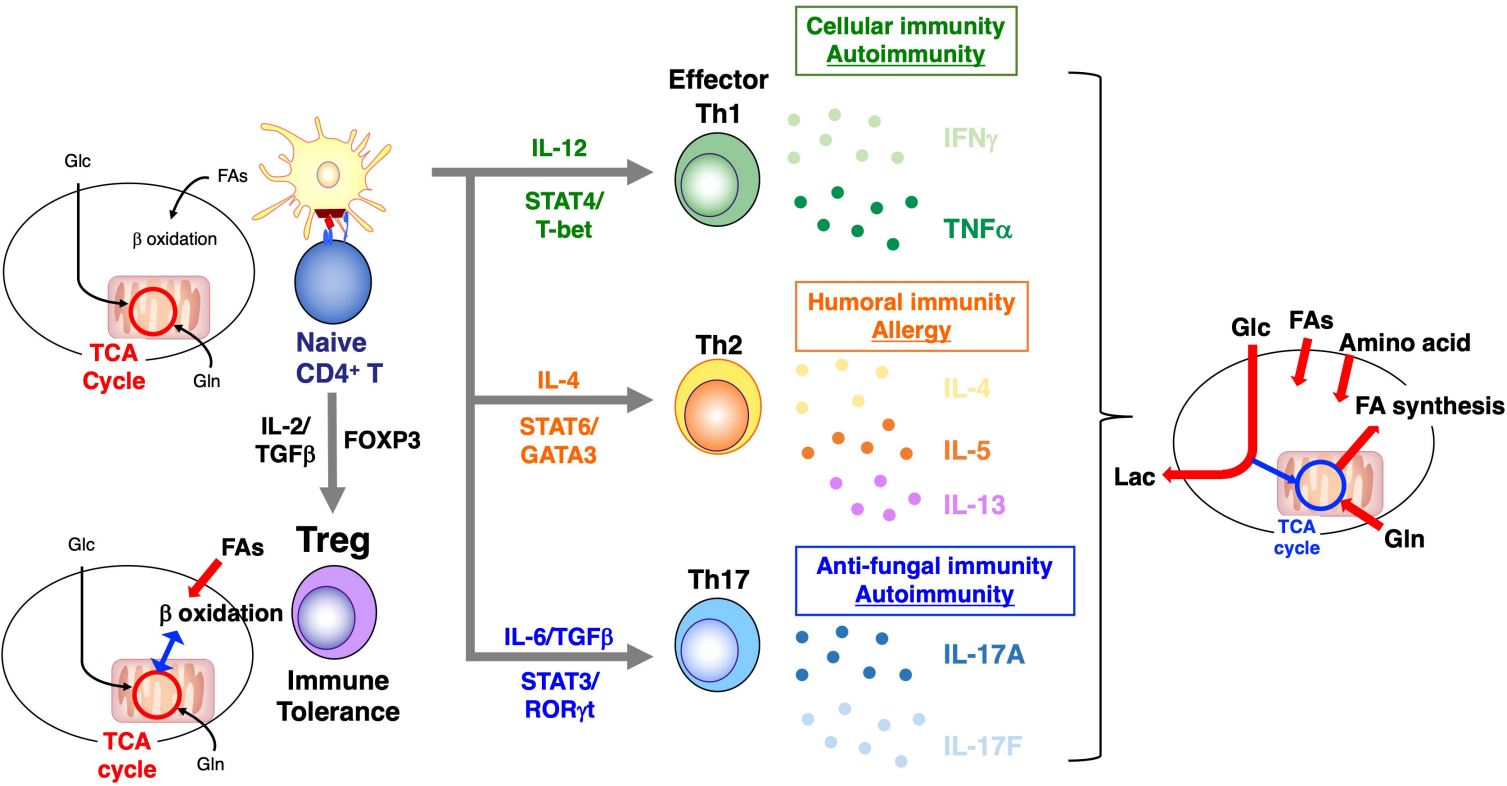
Summary of metabolic profiles of each differentiation stage of T cells. Resting naïve T cells appear to efficiently but very slowly produce ATP by oxidation of glucose-derived pyruvate along with lipids in TCA cycle and OXPHOS pathway. In contrast, after antigen receptor stimulation, glycolysis and glutaminolysis are increased in order to produce biosynthetic precursors required for rapid cell growth and proliferation in effector phase. At the end of an immune response, effector T cells that survive to become memory T cells go back to resting metabolic status for efficient energy generation.

**Figure 4:**
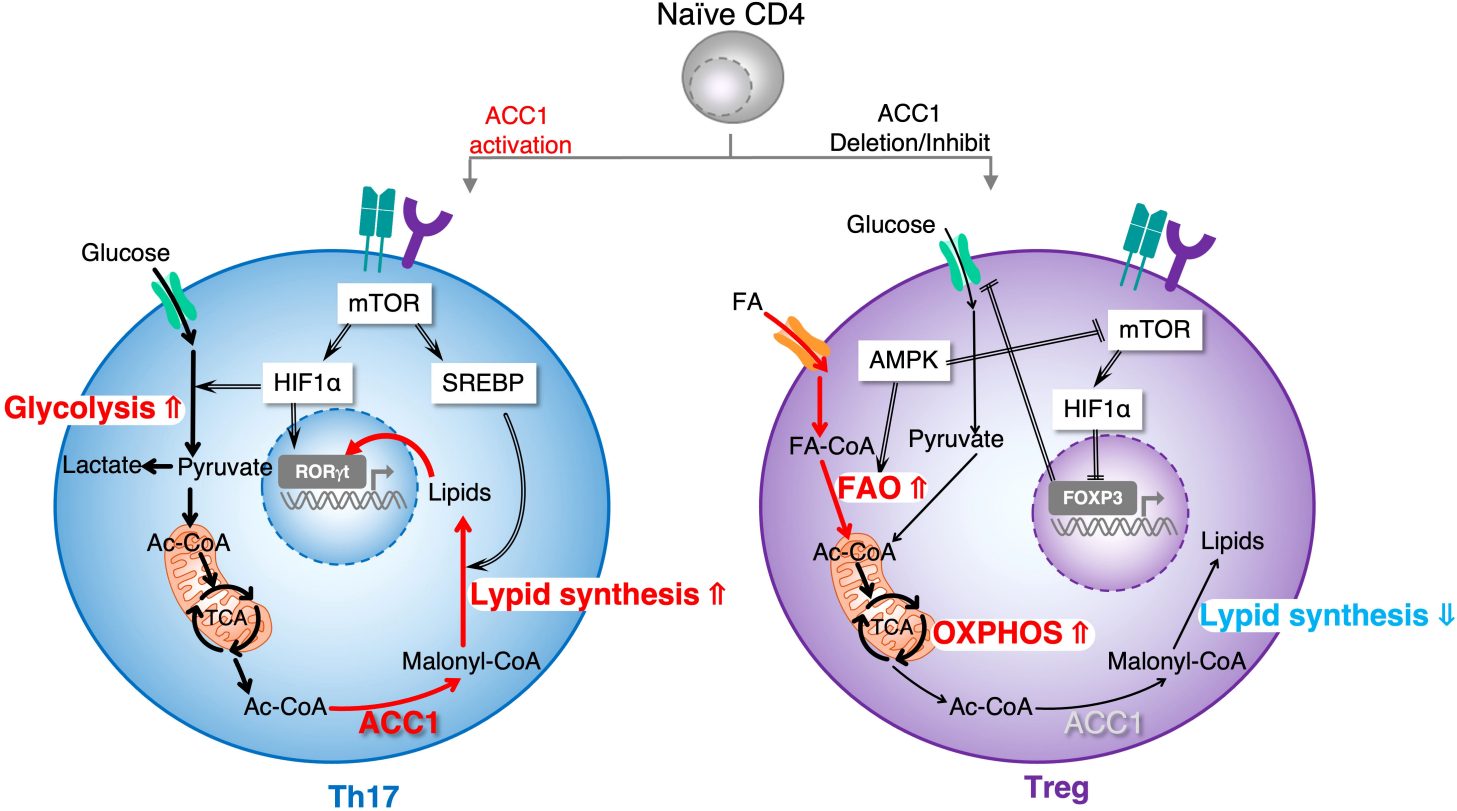
Lipid-driven Th17/Treg cell fate decision. Th17 and Treg cells exhibit reciprocal metabolic programs that determine their lineage specification. Th17 cells rely primarily on *de novo* FA biosynthesis and glycolysis, whereas Treg cells depend on FAO. The mTOR signaling pathway integrates environmental cues to regulate these metabolic states through its downstream effectors, SREBPs and HIF1α. SREBPs drive lipid biosynthesis via the induction of ACC1 and downstream enzymes, while HIF1α enhances glycolytic flux and supports fatty acid synthesis, thereby promoting Th17 cell differentiation. HIF1α also acts as a transcriptional regulator by activating RORγt target genes and facilitating Foxp3 degradation, shifting the balance toward Th17 fate. In contrast, AMPK counteracts mTOR activity by promoting catabolic processes such as FAO and autophagy, thereby favoring Treg differentiation and maintaining Foxp3 stability.

Th cell differentiation from naïve T cells is controlled by the cytokine microenvironment. As with T cell activation, metabolic control is also necessary for Th subset differentiation (55). It is widely accepted that Th1, Th2, and Th17 effector cells are highly dependent on glycolysis, whereas Treg cells preferentially utilize FAO (56) (**Figure 3**). In addition, the specific function of Th1 cells is highly dependent on the environmental levels of FA. For example, long-chain fatty acids, including capric acid (C10:0) and lauric acid (C12:0), enhance Th1 cell differentiation via the p38-MAPK pathway, exacerbating the disease state in experimental autoimmune encephalomyelitis (EAE) mice (57). In contrast, polyunsaturated FAs (PUFAs) such as docosahexaenoic acid downregulate the cytokine expression associated with Th1 cells (58). As already noted, Treg cells predominantly use mitochondrial FAO for their development and survival, whereas Th17 cell differentiation is heavily dependent on *de novo* FA biosynthesis (37, 59). Th17 cells cultured with IL-23 and IL-1β showed a more inflammatory phenotype, resulting in the upregulation of the genes involved in FA biosynthesis (60). The inhibition of FA biosynthesis, either by the pharmacological inhibitor TOFA or by causing a deficiency of ACC1, specifically in CD4^+^ T cells, significantly inhibits Th17-cell differentiation (37). Similarly, the proportion and number of IL-17A–producing memory phenotype CD4^+^ T cells have also been shown to decrease in mice deficient in ACC1. The reciprocal metabolic programs of Th17 and Treg cells are driven primarily by glycolysis, fatty acid biosynthesis and fatty acid oxidation (FAO) (4). In detail, the downstream effectors of mTOR include SREBPs and HIF1α. SREBPs activate transcriptional networks that promote lipid biosynthesis, whereas HIF1α orchestrates glycolytic metabolism to supply carbon substrates for fatty acid synthesis and directly contributes to the transcriptional program of effector T cells. Notably, T cell–specific HIF1α or ACC1 deficiency results in impaired Th17 differentiation but enhanced Treg development (37, 59, 61). Therefore, HIF1α-induced glycolysis and ACC1-mediated lipogenesis programs function as a metabolic switch governing the Th17/Treg balance by transactivating RORγt target genes. Conversely, AMP-activated protein kinase (AMPK) inhibits anabolic pathways and activates catabolic processes such as FAO and autophagy to restore energy homeostasis. By suppressing mTOR signaling, AMPK establishes a metabolic milieu that disfavors Th17 differentiation while promoting Foxp3^+^ Treg stability (**Figure 4**). Furthermore, recent studies have highlighted the critical role of the lipid metabolic pathways in Th17 cells, and lysophosphoethanolamine (LPE) has been identified as a key factor (62). Comprehensive lipidomic analyses demonstrated that LPE (1-18:1) enhanced Th17 cell differentiation by regulating RORγt, which is crucial for the development of T cells, especially Th17 cells. Furthermore, the genetic deletion of *Pla2g12a*, a gene responsible for LPE biosynthesis, impaired Th17 cell differentiation and abolished the EAE pathology.

Recent RNA sequencing studies performed using T cells from EAE mice have demonstrated that low levels of CD5 molecules like (CD5L) are correlated with Th17 pathogenicity (63, 64). CD5L regulates FA flux through the *de novo* FA biosynthetic pathway (63, 64). Lipidomic analyses in CD5L-deficient Th17 cells revealed global differences in the FA composition of phospholipids and neutral lipids. Analyses of CD5L-deficient Th17 cells revealed increased levels of saturated FAs and monounsaturated FAs (MUFAs), and a corresponding decrease in PUFAs, such as C20:4 (64). Furthermore, the exogenous addition of PUFA to Th17 cells decreased the binding of RORγt at *Il17* and *Il23r* loci, whereas providing exogenous saturated FAs or MUFAs increased RORγt binding to the target gene loci (64). Thus, the intracellular FA composition appears to affect the Th17 function, at least in part, via RORγt recruitment and/or activity at the signature cytokine loci.

Advances in the lipid metabolite analysis methods have revealed that the degree of FA saturation plays a key regulatory role in the T cell function (65, 66). For example, linoleic acid (LA; C18:2) has been shown to enhance cytotoxic the T lymphocyte (CTL) function and memory cell formation through mitochondrial reprogramming. Exogenous supplementation with C18:2 improves both the quality and quantity of mitochondria, thereby preventing CTL exhaustion and enhancing antitumor responses (65). In contrast, within the tumor microenvironment, endogenous FA biosynthesis in CTLs disrupts their bioenergetic fitness. Tumor-infiltrating T cells (TILs) accumulate excessive lipid droplets (LDs), which impair the full activation of antitumor responses. This accumulation is mediated by ACC1, leading to enrichment of the saturated fatty acid pool, particularly palmitic acid (C16:0) and stearic acid (C18:0). The pharmacological inhibition of ACC promotes FAO, thereby enhancing CD8^+^ TIL persistence and improving tumor control (66). In addition, our studies have shown that the MUFA metabolism controls antiviral responses in T cells. Fluctuations in the MUFA metabolism elicit the cGAS-STING pathway for cytosolic DNA sensing, thereby enhancing type I interferon (IFN-I) production. Combinations of CRISPR-based gene editing and untargeted lipidomics analysis methods revealed that stearyl-CoA desaturase 2 (SCD2) is a key regulator of cGAS–STING activation (40). Following STING activation, members of the interferon regulatory factor (IRF) family coordinately regulate IFN-I responses. The deletion of *Scd2* caused IRF3-dependent, but not IRF7-dependent IFN-I production. IFN-I induces IRF9, which directly binds to the transcriptional sites of a wide range of antiviral genes. Importantly, the genetic deletion of *Fads2*, a PUFA desaturase, had minimal effects on IFN-I responses (41).

Recent studies have demonstrated that mitochondrial FAO plays an essential role in the suppressive function of Treg cells in the tumor microenvironment (67). Consistently, another recent study has shown that Tregs rely on FAO and OXPHOS for their homeostasis and suppressive capacity (68). FAO involves the degradation of FAs by the sequential removal of two-carbon units from the acyl chain to produce Ac-CoA, which then enters the mitochondrial tricarboxylic acid cycle to regulate OXPHOS (69, 70). Our recent data show that lung Treg cells effectively acquire exogenous FAs to fuel OXPHOS and that Acsbg1-mediated acyl-CoA synthesis serves as a novel metabolic checkpoint controlling Treg cell homeostasis (70). Indeed, the genetic deletion of *Acsbg1* causes mitochondrial dysfunction in Treg cells. Carnitine palmitoyltransferase 1 (CPT1) present in the outer mitochondrial membrane catalyzes the esterification of long-chain fatty acids with carnitine to form acyl-carnitine (69). The rate of FAO depends greatly on the entry of acyl groups into the mitochondria and thus mainly depends on the activity of CPT1. Consistent with the above findings, Treg development was abrogated by treatment with etomoxir, an inhibitor of CPT1, suggesting that Treg cells depend on this pathway for their development (38).

**Figure 5:**
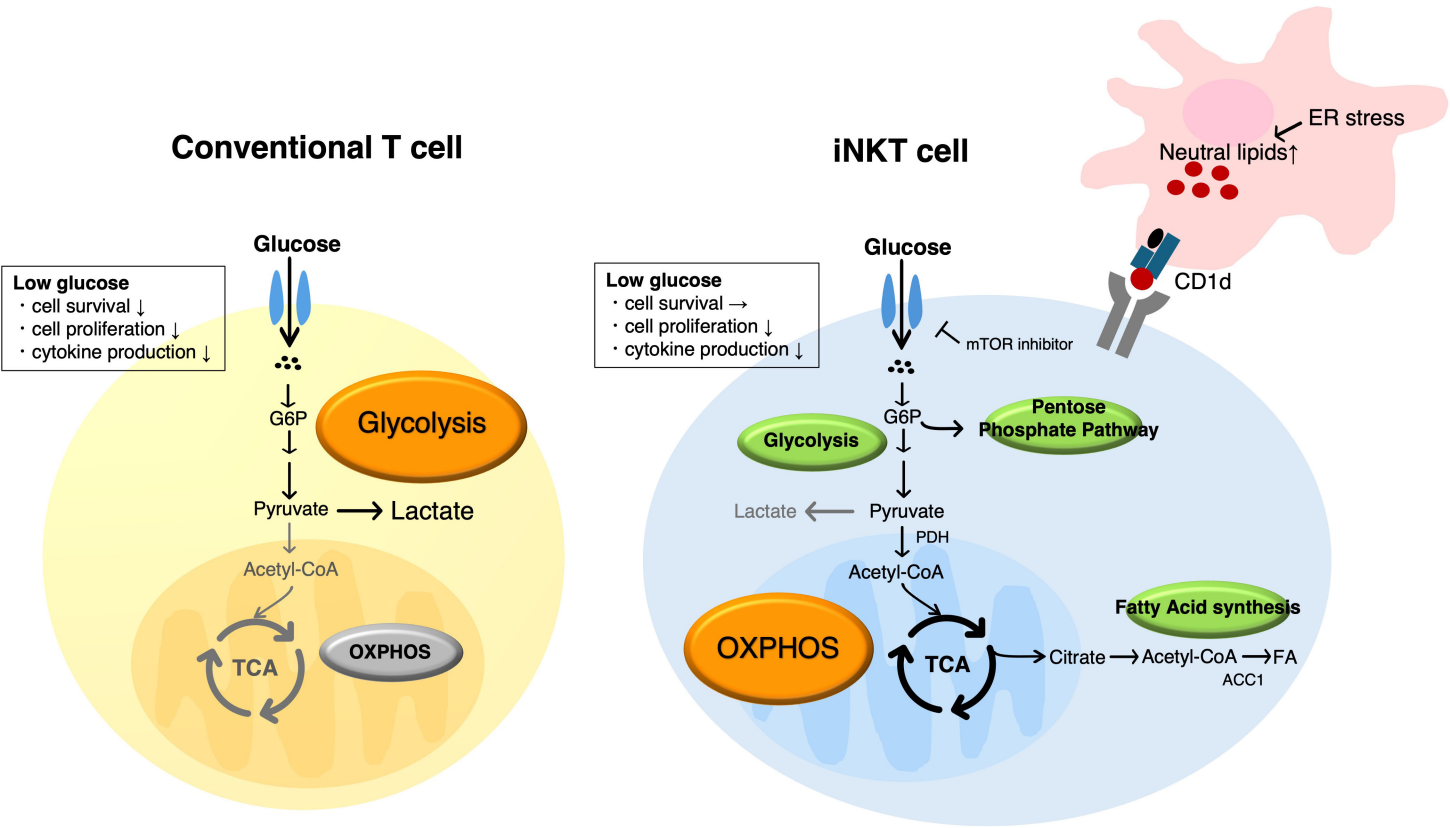
Distinct metabolic remodeling of conventional T cells and iNKT cells during activation. The metabolism of iNKT cells is characterized by a predominance of the pentose phosphate pathway (PPP) and oxidative phosphorylation (OXPHOS), and during activation-induced proliferation, they depend on the activation of mitochondrial metabolic pathways rather than glycolysis.

The lipid metabolism has complex context-dependent effects on iNKT cell development and function. iNKT cells are a lineage of T cells that recognize lipid antigens presented by CD1d molecules, and have innate-like rapid effector functions, including the clearance of infectious organisms, including bacteria and viruses, and elimination of tumor (71, 72). Unlike conventional T cells, which utilize a highly diverse TCR repertoire, iNKT cells possess a semi-invariant TCR composed of an invariant α-chain (Vα14-Jα18 in mice; Vα24-Jα18 in humans) paired with limited β-chains (71, 72). iNKT cells exhibit distinct metabolic features in comparison to conventional T cells, most notably regarding the glucose metabolism. Within the thymus, immature iNKT cells exhibit the elevated expression of the glucose transporter Glut1 and demonstrate robust uptake of glucose. This glucose dependency progressively diminishes as cells mature from stages 0 to 3. The observed correlation between the expression of Glut1 and the proliferation marker Ki-67 further suggests that the glucose demand is particularly high during the immature proliferative phase (73). Even in mature iNKT cells, the glucose uptake is enhanced by TCR stimulation. However, compared with CD4^+^ T cells, iNKT cells exhibit only a modest increase in the expression of Glut1 and the glucose uptake upon activation. Moreover, glucose deprivation had a relatively limited impact on iNKT cell survival, while the production of effector cytokines such as IL-4, IFN-γ, and IL-17 was substantially reduced (74). Thus, although the glucose uptake is enhanced upon activation, iNKT cells appear to rely more heavily on mitochondrial OXPHOS than on glycolysis for energy metabolism. This metabolic preference is supported by the upregulation of genes encoding pyruvate dehydrogenase (PDH) and TCA cycle enzymes in activated iNKT cells (75, 76). In addition, metabolic profiling revealed the accumulation of glucose-6-phosphate (G6P) and intermediates of the pentose phosphate pathway (PPP), thus suggesting that the glucose metabolism in iNKT cells is channeled into multiple pathways beyond glycolysis (**Figure 5**) (74). These findings indicate that glucose metabolism plays an important role in iNKT cell maturation and activation; however, increasing evidence has also highlighted the essential contribution of lipid metabolism to their development and function. Kanno et al. examined mice with a T cell–specific ACC1 deletion (*Acc1*^ΔT^) (77). Strikingly, there was a robust reduction in the number of thymic *Acc1*^ΔT^ iNKT cells, with developmental arrest at the early stages (stage 0/1) and a marked loss of the NKT1 subset. In mixed bone marrow chimera experiments, ACC1-deficient thymocytes failed to compete effectively with their wild-type counterparts in occupying the iNKT cell niche, indicating a cell-intrinsic defect. These findings establish that ACC1 is essential for iNKT cell development, particularly for NKT1 lineage maturation. Although iNKT cells typically receive critical survival signals from cytokines, such as IL-15, during development (78), supplementation with exogenous IL-15 did not rescue the survival defect of ACC1-deficient iNKT cells. This indicates that ACC1 supports iNKT cell development in a cell-intrinsic manner that cannot be compensated for by external growth factors. Combination of the molecular and scRNA-seq analysis showed that ACC1-deficient iNKT cells exhibit the transcriptional hallmarks of metabolic stress and apoptosis. Collectively, these results identify *de novo* fatty acid biosynthesis as a requisite for both anabolic demands (e.g., membrane biogenesis during rapid expansion) and bioenergetic stability (mitochondrial fitness) of developing iNKT cells.

Thus, metabolic regulation plays a pivotal role in the maturation and function of iNKT cells, and a comprehensive understanding of lipid metabolism has become indispensable for elucidating their immunological properties.

## Lipid metabolism in memory T cell generation

Immunological memory is a hallmark of adaptive immunity, wherein the magnitude and efficacy of the response are largely determined by antigen-specific memory T cells. Therefore, clarifying the molecular mechanisms that regulate memory T cell formation holds significant promise for the design of next-generation vaccines and immunotherapeutics (79, 80). Several metabolic approaches have demonstrated that memory T cells, resembling their naïve T cell counterparts, predominantly rely on FAO and OXPHOS to fulfill their energy requirements (81, 82). At the molecular level, the transition toward memory T cells from effector cells is characterized by a metabolic shift from anabolism, such as glycolysis, to fatty acid catabolism via FAO. This metabolic reprogramming, marked by downregulation of glycolysis and upregulation of FAO, is crucial for the generation and long-term maintenance of memory T cells (56, 82–84). Notably, pharmacological inhibition of glycolysis has been shown to enhance memory CD8^+^ T cell formation, suggesting that the extent of glycolytic activity during the effector phase functions as a metabolic rheostat that fine-tunes effector or memory T cell fate decisions (85).

Different types of memory T cells that rely on OXPHOS and FAO preferentially utilize distinct metabolic substrates (86). For instance, *in vitro*-generated IL-15-cultured CD8^+^ T cells with the character of T_CM_ have been shown to depend on lipids synthesized *de novo* from glucose metabolism (82). These cells exhibit reduced fatty acid uptake and lower surface expression of the fatty acid transporter CD36 than effector T cells and Trm cells (87). In contrast, Trm cells are capable of acquiring greater amounts of exogenous fatty acids directly from their local microenvironment and preferentially upregulate lipid chaperones, such as FABP4/5 and CD36, to facilitate this process (87). Interestingly, Berod et al. reported that FAO of long-chain fatty acids is not strictly required for T_CM_ cell formation, emphasizing the ongoing debate regarding the precise metabolic substrates that fuel memory generation (69). Recent findings by Lauson et al. demonstrated that, in both humans and mice, exogenous LA, a polyunsaturated fatty acid, can further enhance the metabolic fitness of human CD8^+^ T cells and promote antitumor immunity (65). LA treatment has been shown to promote ER–mitochondrial contact, thereby enhancing calcium signaling and the mitochondrial energy metabolism. CD8^+^ T cells are activated in the presence of LA-induced lipid remodeling and storage, thereby promoting their differentiation into a memory phenotype. Importantly, these results suggest that in addition to intrinsic metabolic programs such as FAO and OXPHOS, the availability of specific exogenous lipids may regulate the metabolic state of memory T cells. There is no doubt that the FAO/OXPHOS pathway plays an essential role in the formation of memory T cells, but further investigation is required to determine which metabolic pathway is the source of carbon. Upon a secondary antigen encounter, memory CD8^+^ T cells rapidly transition from a quiescent to an active state to initiate a recall response. During this phase, intracellular glycogen serves as a major carbon source, fueling glycolysis and the pentose phosphate pathway via PYGB-mediated glycogenolysis, which is activated through the TCR-associated kinases LCK and ZAP70. This metabolic program supports rapid energy production and antioxidant maintenance before exogenous glucose uptake becomes prominent (88).

**Figure 6:**
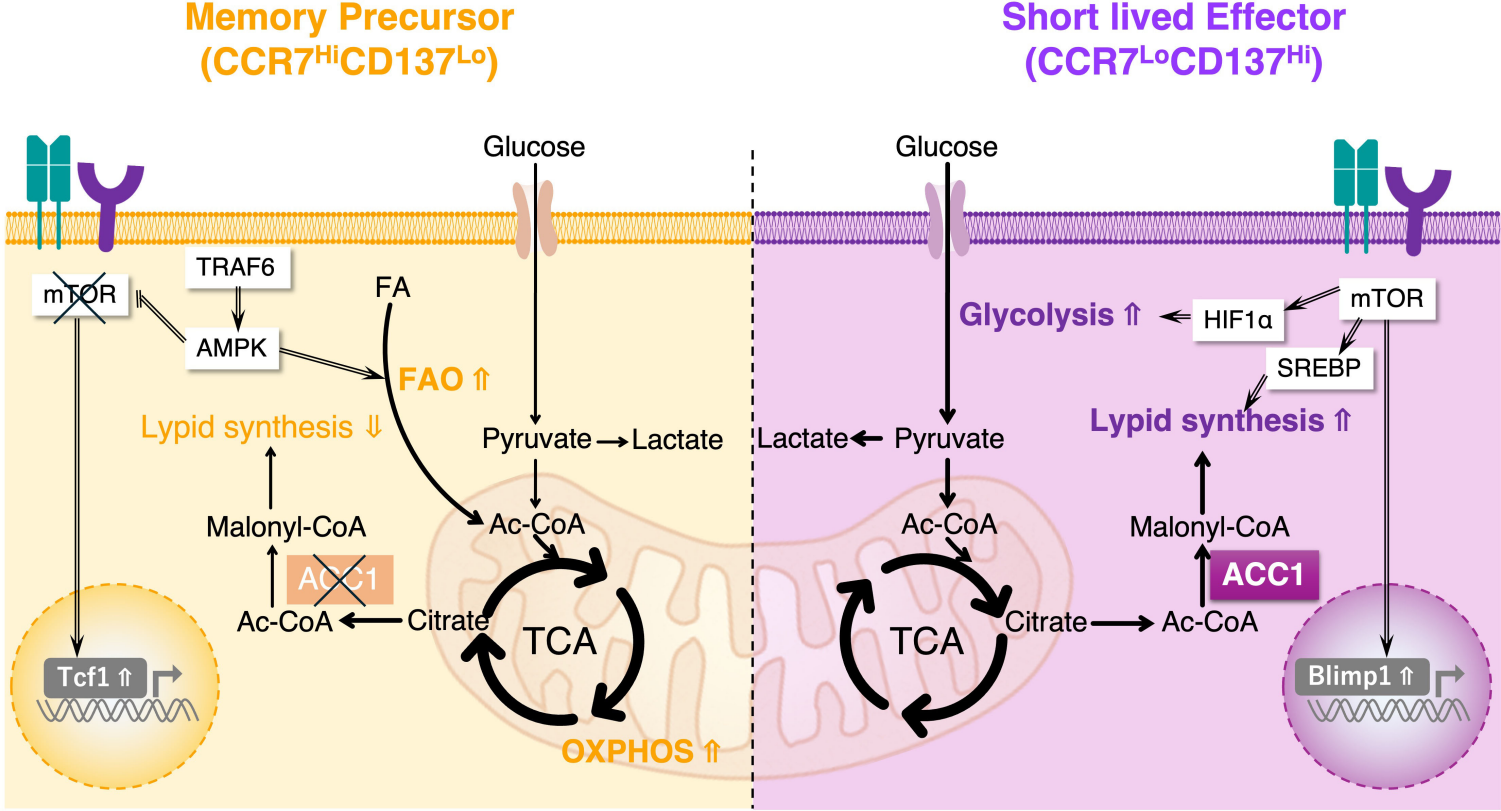
Metabolic reprogramming governs memory T cell fate decision via ACC1-mediated lipogenesis. ACC1 functions as a metabolic switch that regulates the differentiation of T cells into either short-lived effector T cells or long-lived memory T cells. By promoting *de novo* fatty acid synthesis, ACC1 supports the anabolic demands of rapidly proliferating effector T cells. In contrast, genetic deletion or pharmacological inhibition of ACC1 disrupts this lipogenic program and induces a metabolic shift from glycolysis to OXPHOS. This shift favors mitochondrial fitness and promotes the development of metabolically quiescent memory CD4^+^ T cells.

As discussed above, the lipid metabolism plays a crucial role in determining whether cells differentiate into short-lived effectors or long-lived memory cells. A pivotal study demonstrated that ACC1 acts as a metabolic switch that governs fate decisions in both human and murine systems (13). In the experiments, the genetic deletion of ACC1 in CD4^+^ T cells or the acute pharmacological inhibition of ACC1 during the effector phase markedly enhanced the generation of antigen-specific memory T cells, without notably affecting initial T cell activation. Remarkably, ACC1-deficient effector T cells exhibit transcriptional and metabolic signatures resembling memory T cells. For example, they expressed lower levels of lipogenesis-associated genes, including *Acc1*, *Acsl3*, *Mcat* and *Fads2* and higher levels of memory-associated genes, such as *Klf2*, *Ccr7*, *Foxo1*, and *Tcf7*, aligning with a more memory-prone state. Consistent with these observations, single-cell analyses further demonstrated that early effector T cells could be clearly divided into two distinct populations based on the expression of *Acc1* gene. Importantly, the ACC1^lo^ early effector T cell population expresses genes associated with a memory-like transcriptional signature. In contrast, the effector T cell population with higher *Acc1* expression levels displays a short-lived, terminally differentiated phenotype. Furthermore, the segregation of the CCR7 and CD137 expression within early effector CD4^+^ T cells is closely linked to distinct metabolic profiles, transcriptional programs, and eventual cell-fate decisions. Notably, the majority of naïve CD4^+^ T cells differentiate into an ACC1^hi^CCR7^lo^CD137^hi^ effector cell population, characterized by elevated FA biosynthesis, reduced mitochondrial respiratory capacity, a Blimp1^hi^TCF1^lo^ transcriptional pattern, and limited cell longevity, presumably due to an impaired metabolic transition from an activated to a quiescent state during the contraction phase. Conversely, the less abundant ACC1^lo^CCR7^hi^CD137^lo^ effector population exhibited low FA biosynthesis, high mitochondrial respiratory capacity, and a Blimp1^lo^TCF1^hi^ profile, enabling long-term survival *in vivo* (**Figure 6**).

Mechanistically, ACC1–mediated FA biosynthesis appears to favor terminal effector T cell differentiation at the expense of memory cell formation. When ACC1 is active, T cells utilize acetyl-CoA for fatty acid biosynthesis, supporting both rapid cell growth and the effector functions (e.g., building membranes for proliferating cells and supplying lipid mediators for inflammatory signaling) (37, 59). This comes at a cost: high lipogenesis and glycolysis can lead to harmful metabolite accumulation and oxidative stress, which may shorten the lifespan of cells (89). In ACC1-deficient T cells, however, reduced lipogenesis which forces cells to rely more on fatty acid catabolism and mitochondrial oxidative metabolism, a metabolic profile that is associated with greater stress resistance and longevity (metabolic characteristics of memory T cells). Supporting this, ACC1-deficient T cells showed increased mitochondrial membrane potential and resistance to apoptosis, traits that are favorable for long-term survival. Importantly, blocking ACC1 during the early effector phase (e.g., with 5-tetradecyloxy-2-furoic acid: TOFA) did not prevent immediate effector function of T cells, such as cytokine production (IL-4 or IFN-γ). Thus, ACC1 functions as a critical metabolic checkpoint, directing T cell fate decisions, and its activation favors a metabolically demanding effector program with limited cell longevity, whereas its inhibition promotes an energetically efficient metabolic state suitable for the formation of memory cells. In line with this mechanistic framework, recent studies have shown that ACC1 activity obstructs lipid utilization by tumor-infiltrating CD8^+^ TILs (66). Indeed, restricting ACC1 activity reprogrammed TIL metabolism toward fatty acid oxidation and mitochondrial respiration. Notably, such metabolic rewiring was accompanied by transcriptional and phenotypic programs that promoted T cell persistence, ultimately supporting sustained antitumor responses. Interestingly, the role of ACC1 appears to be age dependent. Ibitokou et al. reported that ACC1 deletion in *Cd4*^cre^*Acc1*^fl/fl^ mice during the early stages of naïve CD4^+^ T cell activation in a chronic *Plasmodium chabaudi* infection model significantly impaired the generation of memory precursor T cells, indicating that ACC1 is essential for memory precursor formation following primary immunization (90). In contrast, Endo et al. showed that ACC1 inhibition in differentiated effector CD4^+^ T cells promotes the formation of primary memory T cells, and further assessed its impact after reimmunization using a reinfection model. Together, these findings suggest that the ACC1-mediated fatty acid metabolism plays a stage-specific role in T cell differentiation, which is critical for memory precursor formation during the early phase, whereas at later stages, it reinforces effector fate and limits cell lifespan, such that its inhibition may enhance memory T cell development. This paradigm highlights a compelling opportunity to enhance immune memory through transient modulation of metabolic enzymes during the course of an immune response.

The lipid metabolism modulates memory T cell formation not only through cytosolic fatty acid synthesis, but also via organelle-specific metabolic pathways. In a recent study, Steiner et al. investigated the role of mitochondrial fatty acid synthesis (mtFAS), a metabolic pathway that generates mitochondrial lipids independent of the conventional cytosolic FAS pathway (91). Through CRISPR/Cas9-based screening of 47 lipid metabolism–related genes, they identified mitochondrial trans-2-enoyl-CoA reductase (MECR) as a critical regulator of T cell lipid metabolism *in vivo*. MECR catalyzes the terminal step of mtFAS and produces the acyl-acyl carrier protein (acyl-ACP), a key intermediate in mitochondrial lipid biosynthesis. Functional analyses using *Cd4*^cre^*Mecr*^fl/fl^ mice accumulated mitochondrial reactive oxygen species (mtROS), thereby compromising their functional capacity and survival. While resting T cell populations appeared unaffected by MECR loss, activated MECR-deficient T cells displayed markedly impaired proliferative capacity and yielded fewer effector (CD62L^-^CD44^+^) and T_CM_ (CD62L^+^CD44^+^) T cell subsets *in vivo*. These findings underscore that mtFAS via MECR is essential for T cell proliferative expansion and proper execution of both effector and memory differentiation programs. Collectively, this study highlights the essential role of mtFAS in maintaining mitochondrial integrity and sustaining metabolic fitness required for long-term T cell-mediated immunity.

## Conclusion

In conclusion, the lipid metabolism has emerged as a central multifaceted regulator of CD4^+^ T cell biology. The early induction of *de novo* fatty acid synthesis facilitates effector differentiation, whereas subsequent metabolic reprogramming supports the generation and persistence of memory T cells. Moreover, the subset-specialized lipid requirements of Th17, Treg, and iNKT cells (metabolic processes) are deeply involved in the fate and function of immune cells. Rather than serving as a passive background process, the metabolism actively instructs T cell fate decisions, operating in concert with canonical transcriptional networks. Advances in integrative multi-omics approaches have begun to map this metabolic circuitry with unprecedented resolution, revealing critical checkpoints such as ACC1-mediated fatty acid synthesis and sphingolipid biosynthesis, which can be manipulated to fine-tune immunity. and sphingolipid biosynthesis, which can be manipulated to fine-tune immunity.

Future studies integrating transcriptomic, metabolomic, and spatial lipidomic analyses will further elucidate the temporal and spatial dynamics of metabolic control in T cell differentiation. Such approaches will provide a systems-level understanding of how distinct metabolic states shape immune cell identity and function. These insights not only advance our fundamental understanding of the immunometabolism, but also guide the development of novel strategies to control the immune responses in disease and therapy.
